# Frequency and management of emergencies in primary care offices: A cross-sectional study in northwestern Germany

**DOI:** 10.1080/13814788.2022.2094912

**Published:** 2022-07-12

**Authors:** Max Melzel, Falk Hoffmann, Michael H. Freitag, Ove Spreckelsen

**Affiliations:** a Division of General Practice, Department of Health Services Research, Carl von Ossietzky University of Oldenburg, Oldenburg, Germany; b Division of Outpatient Care and Pharmacoepidemiology, Department of Health Services Research, Carl von Ossietzky University of Oldenburg, Oldenburg, Germany

**Keywords:** Multivariate analysis, incl. modelling, surveys, emergency and out-of-hours care, general practice/family medicine, general, medical education

## Abstract

**Background:**

Little literature exists on emergencies within primary care offices.

**Objectives:**

We aimed to study the occurrence of emergencies and confidence in dealing with them among primary care physicians (PCPs) in Germany.

**Methods:**

We conducted a cross-sectional study among all PCPs with licences to practice with an own office (*n* = 915) in a northwestern region in Germany in 2019. Participants were asked to estimate the frequency and type of emergencies that occurred in the last 12 months in their office and about their confidence in managing emergency situations.

**Results:**

Answers from 375 PCPs could be analysed (response: 41.0%); 95.7% reported at least one emergency in their office within the last 12 months (mean 12.9). PCPs from rural offices reported more emergencies (on average 13.7 vs. 9.6). Acute coronary syndrome, cardiac arrhythmia and dyspnoea were the most common emergencies. A greater likelihood of feeling more confident in managing medical emergencies was found among male physicians, general internists, PCPs additionally qualified as emergency physicians and those with previous training in the emergency department and intensive care unit. In contrast, more general practitioners felt secure treating paediatric emergencies than general internists (highest level of confidence 22.1% vs. 16.3%).

**Conclusion:**

In Germany, emergencies in primary care offices occur on average once a month and more often in rural than urban areas. While most PCPs are confident in managing medical emergencies, some differences related to the training path became apparent. Ongoing training programmes may be tailored to improve emergency skills.

KEY MESSAGES

- From German primary care offices, more emergencies are reported than internationally.
- Most primary care physicians feel adequately prepared for dealing with emergencies but indicate some insecurities depending on the type of emergency and their qualifications.
- Continuous medical education may be improved when tailored to the physicians’ needs in handling emergencies.

## Introduction

Primary care physicians (PCPs) may be confronted with an emergency that leads to an acute hospital treatment need as they are often the first point of contact to the health care system for patients [1].

KEYMESSAGES

- From German primary care offices are more emergencies reported than internationally.
- Primary care physicians feel generally prepared for dealing with emergencies but indicate some insecurities depending on the type of emergency and their qualifications.
- Continuous medical education may be improved when tailored to the physicians’ needs in handling emergencies.

International literature on the frequency and type of emergencies that occur within primary care offices (PCOs) is sparse and indicates that emergencies are rare but do still occur regularly within an office [2–5]. An Australian study, for instance, found that PCPs experienced an average of seven emergencies per year [2]; 71.0% of American and 84% of Irish PCPs saw an emergency with chest pain (ACS), dyspnoea or a paediatric emergency at least once a year [3,4]. Epileptic seizures, serious injuries, and hypoglycaemia were other common emergencies [4]. A Canadian study showed that two percent of all emergency calls came from PCOs due to life-threatening events [5]. In comparison, a small, conveniently sampled survey from Germany among 128 PCPs and 50 emergency physicians showed similar results with 1–3 emergencies reported per year and office and more often in group than in single offices [6].

The location might also influence the frequency of emergencies, as results from Australia show about 30% more emergencies in PCOs outside metropolitan areas [2].

Emergency situations can pose stress to the office staff, including the physician. Therefore, a structured approach to emergency situations including regular training was suggested earlier [7]. More specifically, confidence in managing emergencies in the office correlates with the frequency of training in those situations [8]. In Germany, less than half of all PCPs participate in emergency training annually, with more frequent participation from rural areas [6]. Overall, few international studies on frequencies and types of emergency situations in PCOs are available and possible urban-rural differences are often not assessed. For Germany, no representative data is available.

Therefore, we aimed to study: (a) how often and what type of emergencies occur in PCOs in Germany; (b) how PCPs and their staff prepare for emergency situations; (c) how PCPs assess their confidence in dealing with various emergencies; (d) whether there are differences between rural and urban offices and according to training pathways of PCPs.

## Methods

### Design and setting

We carried out a cross-sectional study among all PCPs (approximately 915) licenced to practice with own office by the regional association of Statutory Health Insurance Physicians in the northwestern region of Lower Saxony (Germany) with about 1,300,000 inhabitants. The cross-sectional study took place from March until July 2019. All questionnaires were personally addressed by post, including a short cover letter with information about the survey. All questionnaires were returned anonymously. Several strategies shown by a Cochrane review to increase response to postal surveys were applied, including a reminder letter [9].

### Questionnaire and information assessed

A multidisciplinary research team developed a four-page questionnaire (see eTable 3). Emergencies were defined as incidents where, unexpectedly a rescue service or emergency physician service was called for assistance and patient’s hospitalisation followed. For the most common types of emergencies, questions about emergency management and further training were constructed based on the literature and own experience [2–5,10]. External pre-tests from five PCPs were obtained, which led to the final questionnaire.

The questionnaire was divided into six sections. The first section queried the frequency of emergencies in the past 12 months, the third and fourth sections dealt with questions relating to emergency training of the physician and the office team. The fifth section dealt with confidence in managing emergencies and perceived security until the ambulance arrived on a 6-point Likert scale (1 = very secure, 6 = very insecure). In the last section, sociodemographic characteristics of the physician, training pathways and additional qualifications, as well as characteristics of the office (e.g. type of office: single office, group office, medical care centre; location: rural, mixed, urban) and distances to the nearest hospital and rescue station were obtained. For all analyses, the ‘mixed’- location category (subjective assessment of the catchment area as neither clearly rural nor urban) was assigned to rural. A remaining section with questions on emergency equipment and medication will be analysed separately.

To qualify as a PCP in Germany, there are two different training paths: a five-year residency as a general practitioner (GP) with mandatory training in a PCO and a hosptal-based five-year residency in internal medicine. For emergency situations, the internist pathway requires rotations to the intensive care unit and emergency department, whereas for GPs, these are optional [11,12]. Furthermore, physicians can obtain an additional qualification in emergency medicine with an 80-hour emergency medicine course and 20–50 rescue operations under supervision [11].

### Statistical analysis

Descriptive statistics were calculated and presented as proportions (%) for categorical responses and means with standard deviations (SD), medians as well as the range for continuous responses. The data was further stratified according to the aforementioned office and sociodemographic characteristics. Differences were assessed using Student *t*-tests and chi-square tests (χ^2^ test; level of significance *p* < 0.05). We calculated a multiple linear regression model to assess predictors for the frequency of emergency situations within a year. As explanatory variables, the location of the office (rural vs. urban) and the type of office (single vs. group offices, including medical care centres) were included. Both Likert-Scales (five-fold on statements about continuous education related to emergency medicine; six-fold about confidence in handling emergencies) were collapsed into binary variables for further statistical analyses. To obtain predictors for higher confidence in dealing with emergency situations in the waiting time until the emergency service arrives, we fitted a multivariate logistic regression model. We included variables with respect to the qualification of the PCP (GP vs. internist; rotations to the ED or ICU during residency; additional qualification as an emergency physician), sociodemographic characteristics (age, sex) and office characteristics (location, office type) in the model. All analyses were performed with SPSS (IBM SPSS Statistics Version 25) for MacOS.

The study received a waiver (No. 2018-130) by the local medical ethics committee of the Carl von Ossietzky University Oldenburg.

## Results

### Characteristics of the respondents

**Figure 1.**
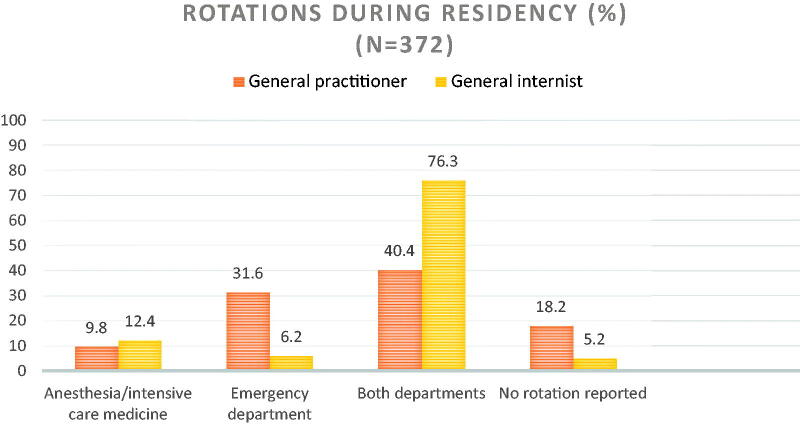
Proportion of completed rotations during residency by training pathway.

A total of 383 of 915 questionnaires were returned. Eight of these were empty, therefore 375 could be analysed (response 41.0%), including six with no information on the office’s location. Most participants were in the age group of 55 years and older and two-thirds were male (Table 1). About half worked in group offices. About three-quarters were trained as GPs and a lesser part of those reported rotations relevant for emergency management than internists (Figure 1). The bulk of GPs (81.4%) and internists (91.7%) rated the relevance of these training phases as important or very important for their work in PCOs.

**Table 1.** Baseline characteristics.

Characteristics	Total [*n* = 375]^b^ (*n*/%)	Rural [*n* = 294]^a^ (*n*/%)	Urban [*n* = 75]^a^ (*n*/%)
Age in years (*n* = 372)			
<45	43 (11.6)	33 (11.3)	10 (13.5)
45–54	126 (34.9)	97 (33.1)	29 (39.2)
55+	203 (54.6)	163 (55.6)	35 (47.3)
Sex (*n* = 371)			
Female	118 (31.8)	86 (29.6)	32 (42.7)
Male	253 (68.2)	205 (70.4)	43 (57.4)
Qualification of the PCPs (*n* = 372)			
General practitioners (GP)	275 (73.9)	220 (75.3)	52 (69.3)
General internists	97 (26.1)	72 (24.7)	23 (30.7)
Time since qualification as a PCP in years (*n* = 371)			
Median	18.2 years (mean 18.0, SD 9.7; range 0.2–43)	18.6 years (mean 18.5, SD 10.0; range 0.2–43	16.2 years (mean 17.0 SD 8.0 range 3–33)
Additional qualification emergency physician (*n* = 371)			
No	185 (49.9)	144 (49.5)	38 (50.7)
Yes	186 (50.1)	147 (50.5)	37 (49.3)
Currently working as an emergency physician (*n* = 372)			
No	339 (91.1)	261 (89.4)	73 (97.3)
Yes	33 (8.9)	31 (10.6)	2 (2.7)
Practice type (*n* = 373)			
Single Primary care office	177 (47.5)	144 (49.1)	30 (40.0)
Group Primary care office	191 (51.2)	145 (49.5)	44 (58.7)
Medical care centre	5 (1.3)	4 (1.4)	1 (1.3)
Rotations during residency (*n* = 373)			
Emergency department	93 (24.9)	75 (25.5)	16 (21.3)
Intensive care medicine/anaesthesiology	40 (10.7)	31 (10.5)	7 (9.3)
Both departments	185 (49.6)	144 (49.0)	40 (53.3)
No rotation	55 (14.8)	43 (14.7)	12 (16.0)
Distances			
Distance to the nearest hospital (*n* = 372)	9.0 km (SD 7.4, range 0–30)	10.7 km (SD: 7.4, range: 0–30)	2.7 km (SD: 2.0, range: 0–12)
Distance to next rescue station (*n* = 370)	5.0 km (SD 4.4, range 0–25)	5.6 km (SD: 4.6, range: 0–25)	2.2 km (SD: 1.6, range: 0–8)

^a^Differences in *n* due to missing values.

^b^Including respondents with no information on the location of their office but answered other questions.

Most respondents came from predominantly rural areas (79.7%) where single offices were more common than in urban areas (49.1% vs. 40.0%). Furthermore, PCPs working in rural areas were older, more often males and were more often qualified as a GP. The distance to the nearest hospital was further from rural offices (mean 10.7 vs. 2.7 km).

### Frequency and type of emergencies

The respondents reported a total of 4499 emergencies (median per PCO: 7.0; mean 12.9, SD: 17.4) for the previous 12 months (4.3% reported no emergency). On average more emergencies were reported from rural offices (mean 13.7, SD 18.7, range 0–120 vs. 9.6, SD 10.9, range 0–62) and from groups compared to single offices (17.3, SD 19.5 vs. 7.2, SD 9.0). After controlling for the office size in the multiple linear regression model, an average of 4.9 more emergencies were reported from rural PCOs (see eTable 1).

Most common emergencies were acute coronary syndrome, followed by arrhythmia, acute dyspnoea, and stroke (Table 2). Serious injuries, (suspected) poisoning, and paediatric emergencies were rare. Emergencies were more often reported from rural than urban PCOs (e.g. three times or more (suspected) acute coronary syndrome: 59.2% vs. 50.0%).

**Table 2.** Reported emergency situations during the past 12 months resulting in a call of the emergency service.

Emergency situation	Frequency in the past 12 months	Total^b^ (*n*^a^/%)	Rural^a^ (*n*/%)	Urban^a^ (*n*/%)	*p*-Value^c^
Acute coronary syndrome (suspected) (*n* = 363)	3 times or more often	209 (57.6)	170 (59.2)	35 (50.0)	0.323
	1- to 2-times	131 (36.1)	101 (35.2)	29 (41.4)	
	never	23 (6.3)	16 (5.6)	6 (8.6)	
Cardiac arrhythmia (*n* = 334)	3 times or more often	125 (37.4)	107 (40.8)	18 (26.9)	0.101
	1- to 2-times	120 (35.9)	90 (34.4)	27 (40.3)	
	never	89 (26.7)	65 (24.8)	22 (32.8)	
Acute shortness of breath/dyspnoea (*n* = 311)	3 times or more often	92 (29.6)	79 (32.5)	12 (19.0)	0.114
	1- to 2-times	110 (35.4)	83 (34.2)	26 (41.3)	
	never	109 (35.1)	81 (33.3)	25 (39.7)	
Stroke (suspected) (*n* = 334)	3 times or more often	89 (26.7)	77 (29.6)	11 (16.7)	0.061
	1- to 2-times	155 (46.4)	122 (46.6)	32 (48.5)	
	never	90 (27.0)	63 (24.0)	23 (34.8)	
Hypertensive urgency (*n* = 310)	3 times or more often	83 (26.8)	73 (29.8)	9 (15.0)	0.039
	1- to 2-times	75 (24.2)	60 (24.5)	14 (23.3)	
	never	152 (49.0)	112 (45.7)	37 (61.7)	
Acute abdomen (suspected) (*n* = 317)	3 times or more often	67 (21.1)	58 (23.2)	8 (12.9)	0.035
	1- to 2-times	110 (34.7)	90 (36.1)	18 (29.0)	
	never	140 (44.2)	101 (40.6)	36 (58.1)	
Psychiatric emergency (*n* = 308)	3 times or more often	35 (11.4)	28 (11.6)	6 (9.5)	0.097
	1- to 2-times	55 (17.9)	49 (20.4)	6 (9.5)	
	never	218 (70.8)	163 (67.9)	51 (81.0)	
Loss of consciousness (*n* = 304)	3 times or more often	28 (9.1)	26 (10.8)	2 (3.2)	0.162
	1- to 2-times	77 (25.0)	58 (24.2)	18 (28.6)	
	never	203 (65.9)	156 (65.0)	43 (68.3)	
Epileptic seizure (suspected) (*n* = 304)	3 times or more often	15 (4.9)	13 (5.4)	2 (3.4)	0.099
	1- to 2-times	44 (14.5)	40 (16.7)	4 (6,7)	
	never	245 (80.6)	186 (77.8)	54 (90.0)	
SIRS/sepsis from febrile infection (suspected) (*n* = 304)	3 times or more often	15 (4.9)	14 (5.9)	1 (1.6)	0.399
	1- to 2-times	66 (21.7)	52 (21.8)	14 (23.0)	
	never	223 (73.4)	172 (72.3)	46 (75.4)	
Paediatric emergency (*n* = 306)	3 times or more often	12 (3.9)	12 (5.0)	0 (0.0)	0.015
	1- to 2-times	25 (8.2)	24 (10.0)	1 (1.6)	
	never	269 (87.9)	203 (84.9)	61 (98.4)	
Hypoglycaemia (*n* = 302)	3 times or more often	10 (3.3)	10 (4.2)	0 (0.0)	0.036
	1- to 2-times	30 (9.9)	28 (11.8)	2 (3.4)	
	never	262 (86.8)	200 (84.0)	57 (96.6)	
Acute anaphylaxis (*n* = 307)	3 times or more often	9 (2.9)	9 (3.7)	0 (0.0)	0.011
	1- to 2-times	65 (21.2)	58 (24.1)	6 (9.8)	
	never	233 (75.9)	174 (72.3)	55 (90.2)	
Injury (severe) (*n* = 305)	3 times or more often	9 (3.0)	9 (3.8)	0 (0.0)	0.112
	1- to 2-times	26 (8.5)	23 (9.7)	3 (4.7)	
	never	270 (88.5)	204 (86.4)	61(95.3)	
Intoxication (suspected) (*n* = 302)	3 times or more often	9 (3.0)	9 (3.8)	0 (0.0)	0.133
	1- to 2-times	26 (8.6)	23 (9.7)	3 (4.9)	
	never	267 (88.4)	204 (86.4)	58 (95.1)	
Cardiopulmonary resuscitation (CPR) (*n* = 301)	3 times or more often	1 (0.3)	1 (0.4)	0 (0.0)	0.370
	1- to 2-times	41 (13.6)	34 (14.5)	5 (8.2)	
	never	259 (86.1)	199 (85.0)	56 (91.8)	

^a^Differences in *n* due to missing values.

^b^Including respondents with no information on the location of their office but answered other questions.

^c^*p*-Values obtained *via* chi-square tests.

### Continuous medical education

An emergency training was attended by 47.0% of physicians and 37.2% of the medical staff at least once a year. Regular emergency training was considered as (very) important for daily practice by most respondents (93.5%).

### Confidence in managing medical emergencies

A majority of both PCPs working in rural and urban areas were confident in handling most emergency situations (Table 3). In contrast, only a minority of PCPs rated themselves as confident when dealing with paediatric and psychiatric emergencies as well as injuries and intoxications. General internists rated themselves more confident than GPs in most emergency situations except paediatric emergencies. One-third rated themselves as moderately or not confident while performing cardiopulmonary resuscitation.

**Table 3.** Proportion of PCPs rating themselves as confident in dealing with specific types of emergency situations.

Emergency situation	Total^b^ (*n*/%)	Rural^a^ (*n*/%)	Urban^a^ (*n*/%)	*p*-Value^c^ (*n*/%)
Acute coronary syndrome (suspected) (*n* = 369)	339 (91.9)	269 (92.4)	65 (89.0)	0.345
Hypertensive urgency (*n* = 371)	357 (96.2)	281 (95.9)	71 (97.3)	0.588
Stroke (suspected) (*n* = 371)	344 (92.7)	270 (92.2)	69 (94.5)	0.488
Hypoglycaemia (*n* = 370)	342 (92.4)	269 (92.1)	68 (93.2)	0.768
Acute abdomen (suspected) (*n* = 371)	339 (91.4)	268 (91.45	67 (91.8)	0.931
Dyspnoea (*n* = 369)	300 (81.3)	235 (80.5)	60 (83.3)	0.580
Epileptic seizure (suspected) (*n* = 368)	293 (79.6)	232 (79.7)	56 (77.8)	0.714
Acute anaphylaxis (*n* = 369)	293 (79.4)	236 (80.8)	53 (73.6)	0.175
*Cardiac* arrhythmia (*n* = 370)	277 (74.9)	216 (74.0)	56 (76.7)	0.630
Cardiopulmonary resuscitation (CPR) (*n* = 370)	250 (67.6)	195 (66.8)	51 (69.9)	0.615
Loss of consciousness (*n* = 369)	232 (62.9)	184 (63.2)	45 (61.6)	0.801
SIRS/sepsis from febrile infection (suspected) (*n* = 368)	218 (59.2)	169 (58.1)	45 (61.6)	0.579
Psychiatric emergency (*n* = 366)	171 (46.7)	132 (45.7)	37 (51.4)	0.384
Injury (severe) (*n* = 367)	149 (40.6)	122 (41.9)	25 (34.7)	0.265
Intoxication (suspected) (*n* = 366)	103 (28.1)	85 (29.4)	16 (22.2)	0.224
Paediatric emergency (*n* = 366)	76 (20.8)	70 (24.1)	6 (8.3)	0.003

^a^Differences in *n* due to missing values.

^b^Including respondents with no information on the location of their office but answered to other questions. Deviations to a total of 100% are due to missing values.

^c^*p*-Values obtained *via* Student´s *t*-tests (two-sided).

PCPs additionally qualified as emergency physicians were most confident in managing most emergencies (see eTable 2 for differences between GPs and internists).

There were no relevant rural-urban differences in the assessment of handling an emergency during the waiting time until the arrival of the rescue service (76.0 vs. 77.0%). A higher chance of feeling confident (Table 4) was associated with an additional qualification as an emergency physician (OR 2.33, CI 1.32–4.11, *p* = 0.003) and a rotation to both the intensive care unit and the emergency department (compared to none) (OR 4.94, CI 2.04–9.91; *p* < 0.001). Only some evidence was found for higher confidence among male physicians (OR 1.67, CI 0.95–2.93; *p* = 0.077) and those working in a group practice (OR 1.75, CI 0.97–3.06; *p* = 0.052).

**Table 4.** Predictors for a higher confidence in dealing with an emergency situation in a PCO during the waiting time until the emergency service arrives (*n* = 353).

Variable	Odds ratio	95%-confidence interval	*p*-Value
Sex			
Female (ref)	1		
Male	1.67	0.95–2.93	0.077
Age			
55+ years (ref)			
45–54 years	1.25	0.68–2.31	0.469
<45 years	1.10	0.40–2.88	0.835
Qualification			
General practitioner (including others) (ref)	1		
Internal medicine	1.64	0.76–3.56	0.210
Rotations during residency			
None reported (ref)	1		
Emergency department only	1.21	0.57–2.53	0.623
Intensive care only	0.94	0.36–2.43	0.940
Both departments	4.94	2.04–9.91	<0.001
Additional qualification in emergency medicine			
Not obtained (ref)	1		
Obtained	2.33	1.32–4.11	0.003
Office localisation			
Rural (ref)	1		
Urban	1.04	0.53–2.06	0.910
Office type			
Single office (ref)			
Group office	1.75	0.97–3.06	0.052

*N* = 353; Pseudo-R2 Cox&Snell 0.158; Nagelkerkes R2 0.237.

## Discussion

### Main findings

#### Frequency and types of emergencies

In our cross-sectional study, an average of 12.9 emergencies per office and year resulting in a call for ambulance service were reported from PCPs from north-western Lower Saxony. This is higher than reported before in Germany and in older international studies [2–6]. We studied a defined larger geographical area, which might explain differences to the smaller, rather older non-representative study from Germany [6]. Regional differences in health and healthcare are known in Germany and might partly explain the higher number of emergencies [13,14]. Furthermore, the increase in multimorbid and older patients due to demographic changes might contribute to increased medical emergencies in the outpatient sector [15]. In comparison to the international literature, the higher number of emergencies in PCOs might also partly be explained by the peculiarities of the German health care system. In international comparison, German GPs have the highest rate of patient contacts per week [16]. This could lead to a higher possibility of an emergency situation within a PCO. The higher number of emergencies in group offices is most likely explained by the larger number of patients seen there compared to single offices.

Higher numbers of emergencies were persistently reported from rural areas. This might be explained by a different sociodemographic structure with an, on average higher age and larger proportion of multimorbidities [17,18]. Furthermore, a lower density of PCOs and an on average higher number of patients in rural areas might contribute to these differences. In rural areas with longer distances to the next hospital, a more closely related doctor-patient relationship may incline patients to consult their PCP first [19]. Furthermore, patients in rural areas tend to present later and with more severe health problems to their PCP, thus possibly leading to higher likelihood of tolerating the development of an urgent health state [20].

#### Confidence

Differences in physicians’ preparedness for an emergency situation could not be found between rural and urban areas. General internists rated their confidence in handling most emergencies slightly higher, while GPs showed higher confidence with paediatric emergencies. Those differences may partly be explained by different proportions of rotations to both the ICU and ED during residency [11]. As a result, internists may have gained more experience in handling emergencies. Rotations and an additional qualification in emergency medicine were associated with greater confidence in dealing with emergency situations. This underlines the importance of continuous medical education focusing on emergency situations. Age and the time since qualification were not associated with a greater confidence in managing emergency situations.

In contrast, more GPs rated themselves as confident in managing paediatric emergencies. However, the relatively large proportion of respondents indicating insecurities suggests that these types of emergencies represent exceptional situations [21].

Equally, feeling uncomfortable when performing cardiopulmonary resuscitation reported by other German GPs could be found among a third of our respondents [22].

In their self-assessment, there was some evidence for higher confidence in dealing with emergencies among men. While male PCPs tend to perform more invasive procedures, we would only cautiously interpret these gender differences as it is known from self-assessments that women tend to underestimate and men tend to overestimate their performance [23,24]. Further studies might therefore assess the performance in emergency situations with more objective parameters.

#### Continuous medical education

About half of the physicians and 37.2% of the medical staff attended annual emergency medicine training courses. Non-regular participation in emergency medicine courses was also evident in other studies [18]. However, most physicians rated emergency training as important to their work as PCP and the availability and content of advanced training courses was rated mainly as satisfactory, with minor differences between GPs and general internists.

### Strengths and limitations

Our study’s main strengths are that we included a well-defined area and were able to receive a response of 41%, which is comparable or even higher than in other studies among GPs [25]. However, more than half of the respondents held an additional qualification in emergency medicine, which is higher than reported in other studies and might indicate non-response bias [18]. Therefore, we potentially found a greater level of confidence in handling emergencies.

The characteristics of the survey area with a rural dominance and no metropolitan areas could limit the generalisability for other parts of Germany. Some PCPs reported a very high number of emergencies in their office, mainly from group offices in rural areas. Those high numbers are possibly explained by PCOs located in holiday areas (coast, East Friesian Islands) where emergencies might occur more frequently during the season. A recall bias could also be possible based on a subjective estimate of the frequency and type of emergencies. The same applies to self-rated confidence in dealing with emergency situations, which might differ from objective measurements of the actual quality of care provided in emergency situations [26]. Furthermore, there were some emergency situations with a comparably high number of missing values (e.g. paediatric emergencies with 20.1%), which might indicate some uncertainty about their frequency.

## Conclusion

In Germany, emergency situations in a PCO occur on average once a month and in rural areas more frequently than in urban areas. Although not confronted with an emergency daily, PCPs from both rural and urban areas, in general, feel confident in managing various emergency situations. However, there were some differences in the type of emergency and training path of the physician. Confidence might be increased with emergency situations that correlate with insecurity as training in paediatric and psychiatric emergencies and regular CPR training. A concept tailored to the needs of PCPs that encourages them to participate regularly in emergency training might increase overall confidence in dealing with emergencies.

## Supplementary Material

Supplemental Material: eTable 3

Supplemental Material: eTable 2

Supplemental Material: eTable 1
