# Supplementary material for: Frequency and management of emergencies in primary care offices: A cross-sectional study in northwestern Germany
Source: Eur J Gen Pract. 2022 Jul 12;28(1):209–16. doi: 10.1080/13814788.2022.2094912 (PMC9291701; doi:10.1080/13814788.2022.2094912)
Supplement: Supplemental Material: eTable 3 [file IGEN_A_2094912_SM3772.docx]

eTable 3. Questions (original questions in German/ authors’ translation to English)

| **No.** | **Frage** | **Question** |
| --- | --- | --- |
| **1** | Bitte schätzen Sie, wie häufig Sie innerhalb der letzten 12 Monate aufgrund eines Notfalls den RTW bzw. Notarzt in Ihre Praxis dazu gerufen haben? | Please estimate how often you have called the rescue service or emergency physicians in your office within the last 12 months due to an emergency? |
| **2** | Wie häufig hatten Sie in den letzten 12 Monaten einen Notfall der folgenden Kategorien in Ihrer Praxis, bei dem Sie den RTW bzw. Notarzt dazu gerufen haben? *(Bitte pro Kategorie nur eine Antwortmöglichkeit markieren)*  A: Kategorien siehe Tabelle 3 | How often have you had an emergency of the following categories in your offices in the last 12 months, for which you called the rescue service or emergency physician? *(Please mark only one answer option per category)*  Answers: For categories, see Table 3 |
| **7b** | Ich bin der Meinung, dass ich durch meine Aus-/Fort- und Weiterbildung ausreichend auf den Umgang mit notfallmedizinischen Medikamenten geschult worden bin  (1= trifft voll zu; 5 = trifft nicht zu) | I believe that I have been adequately trained in dealing with emergency medical drugs through my training and further education  (1 = totally agree; 5 = totally disagree) |
| **10a** | Fortbildungen mit dem Bezug „Akutversorgung von Patienten“ sind für meine hausärztliche Tätigkeit von Bedeutung  (1= trifft voll zu; 5 = trifft nicht zu) | Further training courses related to ‘acute patient care’ are important for my work as a general practitioner  (1 = totally agree; 5 = totally disagree) |
| **10b** | Fortbildungen mit dem Fokus „Akutversorgung von Patienten“ werden ausreichend angeboten  (1= trifft voll zu; 5 = trifft nicht zu) | Training courses with a focus on ‘acute patient care’ are sufficiently available  (1 = totally agree; 5 = totally disagree) |
| **11** | Wie häufig nehmen Sie an einer Fortbildung teil, die sich inhaltlich mit der Thematik der Notfallversorgung von Patienten befasst?  Nie - seltener als alle 2 Jahre/ - alle 2 Jahre/ - 1-mal jährlich – mehr als 1-mal jährlich | How often do you take part in a training course dealing with emergency patient care?  never - less than every 2 years - every 2 years - once a year - more than once a year |
| **12** | Wie häufig nehmen Sie mit Ihrem Praxisteam an speziellen Notfallschulungen teil?  Nie - seltener als alle 2 Jahre - alle 2 Jahre - 1-mal jährlich – mehr als 1-mal jährlich | How often do you and your office team participate in special emergency training courses?  never - less than every 2 years - every 2 years - once a year - more than once a year |
| **13** | Um welche Art von Schulungen handelt es sich?   1. Erste-Hilfe-Kurs 2. Spezieller Kurs für Arztpraxen 3. Fortbildung durch den Arzt selbst 4. andere | In what type of training courses does your office team participate?   1. First aid training 2. courses tailored explicitly for physician offices 3. training by the physicians himself 4. others |
| **16** | Wie sicher fühlen Sie sich in der Behandlung folgender Notfallsituationen?  A: Kategorien siehe Tabelle 3 | How confident do you feel about handling the following emergency situations?  A: For categories see table 3 |
| **17** | Wie sicher fühlen Sie sich generell im Umgang mit Notfallsituationen in der Zeit bis zum Eintreffen des Rettungsdienstes (bzw. Notarztes)?  Likert 1-6 Sehr sicher - unsicher | How secure do you feel in dealing with emergency situations in the time until the arrival of the rescue service (or emergency physicians)?  Likert 1-6 (very secure – insecure) |
| **18** | Angaben zu Ihrer Person/ personal details   1. **Alter/Age:** < 35 Jahre/years – 35-44 Jahr/years – 45-54 Jahr/years – 55-65 Jahr/years - > 65 Jahre/years 2. **Geschlecht /Sex:** weiblich/female männlich/male 3. **Welche Facharztweiterbildung haben Sie absolviert?** *(Mehrfachnennung möglich) /* **What specialist training have you completed***? (Multiple answers possible)*   Allgemeinmedizin/General Practitioner – Innere Medizin/Specialist in general internal medicine – „Praktischer Arzt“ – andere/other   1. **Wie lange sind Sie hausärztlich als Facharzt/praktischer Arzt tätig?/ For how long have you been practicing as a qualified primary care physician (general practitioner/specialist in internal medicine)? Haben Sie einen der folgenden stationären Bereiche in der Weiterbildung zum Facharzt durchlaufen (mindestens 6 Monate)?** *(Mehrfachnennung möglich) /* **Have you completed one of the following inpatient areas as a specialist physician** **(at least for 6 months)***? (Multiple answers possible)* **What departments did you rotate to during your residency?**   Notaufnahme/Emergency department – Intensivstation/Anästhesie (Intensive care station/anesthesia)   1. **Für wie relevant halten Sie diese Anteile in der Aus- und Weiterbildung für die hausärztliche Tätigkeit? / For how important would you rate these rotations during your residency for your work as a general practitioner?** (Likert 1-6) 2. **Haben Sie eine Zusatzweiterbildung im Bereich der Notfall- und Rettungsmedizin absolviert?** *(Notfallmedizin/ alte Bezeichnung Rettungsmedizin) /* **Have you obtained an additional qualification in the field of emergency and rescue medicine?** *(Emergency medicine / old name rescue medicine)* 3. **Fahren Sie neben Ihrer Tätigkeit in der Praxis als Notarzt im Rettungsdienst? / Are you currently working as an emergency physicians?** (Ja/Nein, Yes/No) 4. **In welcher Art von Praxis arbeiten Sie? / In what type of primary care office are you working?**(Einzelpraxis/ single office – Praxisgemeinschaft/ Group office – Medizinisches Versorgungszentrum/ Medical care center) 5. **Wie ist das Einzugsgebiet Ihrer Praxis geprägt? / How is the catchment area of your office characterized?**   Eher ländlich/rural – eher städtisch/urban – gemischt/mixed   1. **Geben Sie die ungefähre Entfernung Ihrer Praxis zum nächsten Krankenhaus mit Notaufnahme an / Please estimate the distance from your office to the nearest hospital with an emergency room** 2. **Geben Sie die ungefähre Entfernung Ihrer Praxis zur nächsten Rettungswache an / Please estimate the distance from your office to the nearest rescue station** | |
| **19** | Anmerkungen/Rückfragen / Comments / queries | |
