# Supplementary material for: Frequency and management of emergencies in primary care offices: A cross-sectional study in northwestern Germany
Source: Eur J Gen Pract. 2022 Jul 12;28(1):209–16. doi: 10.1080/13814788.2022.2094912 (PMC9291701; doi:10.1080/13814788.2022.2094912)
Supplement: Supplemental Material: eTable 2 [file IGEN_A_2094912_SM3679.docx]

eTable 2. Proportion of respondents rating themselves as confident in dealing with emergencies by situation and by qualification

| **Emergency situation** | **Total of confident PCPs (*n*/ %)** | **General Practitioner (GPs) (*n*/ %)** | **General internists**  **(*n*/ %)** | ***P*-value**** |
| --- | --- | --- | --- | --- |
| **Acute coronary syndrome (suspected)**  **(*n* = 369)** | 339 (91.9) | 245 (89.4) | 92 (98.9) | 0.003 |
| **Dyspnoea**  **(*n* = 369)** | 300 (81.3) | 214 (78.4) | 84 (89.4) | 0.188 |
| **Acute abdomen (suspected)**  **(*n* = 371)** | 339 (91.4) | 246 (89.8) | 91 (95.8) | 0.072 |
| **Stroke (suspected)**  **(*n* = 371)** | 344 (92.7) | 250 (91.2) | 92 (96.8) | 0.070 |
| **Hypertensive urgency**  **(*n* = 371)** | 357 (96.2) | 262 (95.6) | 93 (97.9) | 0.317 |
| **Cardiac arrythmia**  **(*n* = 370)** | 277 (74.9) | 195 (71.2) | 80 (85.1) | 0.007 |
| **Sepsis/SIRS**  **(*n* = 368)** | 218 (59.2) | 155 (57.0) | 61 (64.9) | 0.178 |
| **Loss of consciousness**  **(*n* = 369)** | 232 (62.9) | 168 (61.4) | 62 (66.0) | 0.444 |
| **Acute anaphylaxis**  **(*n* = 369)** | 293 (79.4) | 214 (78.1) | 77 (82.8) | 0.334 |
| **Epileptic seizure (suspected)**  **(*n* = 368)** | 293 (79.6) | 213 (78.0) | 78 (83.9) | 0.227 |
| **Psychiatric emergency**  **(*n* = 366)** | 171 (46.7) | 130 (48.0) | 39 (41.9) | 0.314 |
| **Intoxication (suspected)**  **(*n* = 366)** | 103 (28.1) | 66 (24.4) | 35 (37.6) | 0.013 |
| **Paediatric emergency**  **(*n* = 366)** | 76 (20.8) | 60 (22.1) | 15 (16.3) | 0.238 |
| **Hypoglycaemia**  **(n = 370)** | 342 (92.4) | 251 (91.6) | 89 (94.7) | 0.331 |
| **Injury (severe)**  **(*n* = 367)** | 149 (40.6) | 119 (43.4) | 29 (31.9) | 0.051 |
| **Cardiopulmonary resuscitation**  **(*n* = 370)** | 250 (67.6) | 178 (65.0) | 70 (74.5) | 0.089 |

* differences in n due to missing values

** *P*-values obtained via Student´s t-tests (two-sided)
