# Supplementary material for: Frequency and management of emergencies in primary care offices: A cross-sectional study in northwestern Germany
Source: Eur J Gen Pract. 2022 Jul 12;28(1):209–16. doi: 10.1080/13814788.2022.2094912 (PMC9291701; doi:10.1080/13814788.2022.2094912)
Supplement: Supplemental Material: eTable 1 [file IGEN_A_2094912_SM3672.docx]

eTable 1. Emergencies reported within 12 months in PCOs depending on location and practice type (Multiple linear regression analysis), n = 344

| **Variable** | **Estimate** | **95%- Confidence Interval** | **Standard error** | **P value** |
| --- | --- | --- | --- | --- |
| constant | 1.590 | -5.947 – 9.128 | 3.822 | 0.678 |
| Practice location | -4.899 | -9.319 - -0.480 | 2.247 | 0.030 |
| Practice type | 11.242 | 7.716 – 14.769 | 1.793 | < 0.001 |
|  |  |  |  |  |
| R^2^ | 0.111 |  |  |  |
| Corrected R^2^ | 0.106 |  |  |  |
| F (df=2, 343) | 21.312 |  |  | < 0.001 |
